# Notch signaling genes and CD8 ^+^ T‐cell dynamics: Their contribution to immune‐checkpoint inhibitor therapy in oral squamous cell carcinoma: A retrospective study

**DOI:** 10.1002/cam4.6985

**Published:** 2024-03-16

**Authors:** Kazuhiro Ogi, Takahiro Iwamoto, Takashi Sasaya, Koyo Nishiyama, Takaaki Tokura, Takanori Sasaki, Hironari Dehari, Yohei Arihara, Kazuyuki Murase, Masato Saito, Masanori Someya, Kohichi Takada, Akihiro Miyazaki

**Affiliations:** ^1^ Department of Oral Surgery Sapporo Medical University School of Medicine Sapporo Hokkaido Japan; ^2^ Department of Medical Oncology Sapporo Medical University School of Medicine Sapporo Hokkaido Japan; ^3^ Department of Radiology Sapporo Medical University School of Medicine Sapporo Hokkaido Japan

**Keywords:** CD8^+^ T cell, immunotherapy, notch signaling gene, oral squamous cell carcinoma

## Abstract

**Background:**

Aberrant Notch signaling pathway has been related with the tumorigenesis in head and neck region, involving oral cavity. Here, we report the correlation between mutations in the Notch signaling pathway and CD8^+^ T‐cell infiltration via PD‐L1, which lead to enhanced antitumor immunity and may target for immune‐checkpoint inhibitors (ICIs) therapy.

**Methods:**

This retrospective study analyzed the results of immunohistochemical staining for PD‐L1 and CD8^+^ T‐cell infiltration in 10 patients and whole‐exome sequencing (WES) was conducted on five of these patients to identify frequently mutated genes.

**Results:**

Four of 10 patients were positive for PD‐L1 and CD8^+^ T. By analyzing WES in three of these four patients, we notably identified the mutations of NOTCH1, FBXW7, and noncoding RNA intronic mutation in NOTCH2NLR in two of these three patients. This study may enable better selection of ICI therapy with CD8^+^ T‐cell infiltration via PD‐L1 expression for oral squamous cell carcinoma patients with mutations in Notch signaling pathway.

## 1 INTRODUCTION

The Notch signaling pathway fulfills more precious roles in the progression of head and neck squamous cell carcinoma (HNSCC), although the function of Notch signaling is not well understood. The highly conserved Notch signaling pathway modulates a series of fundamental cellular functions, including fateful decision of cell, stemness maintenance, proliferation, and apoptosis.^1^ Mammals have four Notch receptors (Notch 1–4).^2^ The Notch signaling pathway is activated when the ligand expressed on the surface of one cell interacts with the receptor expressed on the plasma membrane of another cell.^2^ In the next step of the interaction with its ligand, the Notch receptor is cleaved by ADAM10/ADAM17 (S2 cleavage) and γ‐secretase complex (S3 cleavage). Subsequently, the Notch intracellular domain (NICD) is released and translocates into the nucleus where it leads to the transcriptional modulation of its target genes. The identification and characterization of the alterations in the NICD underlying individual cancer patients may be critical to develop more effective and personalized therapies.

In HNSCC, recurrence and distant metastasis are facilitated by immune evasion,^3^ which is partially mediated by the expression of programed death receptor ligands 1 and 2 (PD‐L1 and PD‐L2, respectively), which bind programed death 1 (PD‐1), an immune‐checkpoint receptor that suppresses T cells.^4^, ^5^, ^6^, ^7^ Studies elucidating the role of immune evasion by tumor cells have led to several revelations, including the generation of combined positive score (CPS) based on PD‐L1 expression in tumor cells for the assessment of treatment response in HNSCC.

A recent study has found that Notch signaling regulates PD‐1 expression during CD8^+^ T‐cell activation.^8^ However, no study to date has evaluated the role of PD‐L1 expression and CD8^+^ T cell infiltration in aberrant Notch signaling. Moreover, the relationship between aberrant Notch signaling and PD‐L1 expression in the tumor microenvironment remains unclear. PD‐L1 is primarily expressed on the cell membrane and functions as a PD‐1 ligand, whereas some PD‐L1 is also localized in the nucleus.^9^ Although its role in the nucleus is not well known, PD‐L1 is involved in the regulation of gene expression.^10^ Tumor cells with aberrant Notch signaling may acquire unidentified oncogenic functions, leading to the increased expression of nuclear PD‐L1. Tumor cells with high PD‐L1 expression might induce CD8^+^ T cells by promoting the expression of genes involved in inflammation and immune responses, sensitizing tumor cells to anti‐PD‐1/PD‐L1 antibodies. The present study aimed to understand the mechanism underlying the crosstalk between Notch signaling and PD‐L1‐mediated CD8 T^+^ cell infiltration.

In this retrospective study, we hypothesized that the potential relation between mutations in the Notch signaling pathway and tumor immune microenvironment by analyzing CD8^+^ T cells infiltration via PD‐L1, which lead to enhancement of antitumor immunity and confer clinical benefits to immune‐checkpoint inhibitor (ICI) in recurrent or metastatic oral squamous cell carcinoma (OSCC) patients.

## 2 MATERIALS AND METHODS

### 2.1 Data collection and protocol of treatment regimen

A retrospective sub‐cohort study was conducted to genetically profile ctDNA from pretreatment plasma samples of patients and DNA from biopsy tissue. We archived a retrospective analysis on patients with stage II–IV OSCC who were administrated nivolumab or pembrolizumab in second‐ or further‐line therapy from April 2017 to October 2022. We did not collect data on HPV status because of the limited evidence indicating that HPV status has minimal impact on survival or treatment response in oral cancer.

Initially, we included a total of 17 patients in our retrospective study. Nevertheless, we subsequently excluded seven patients from the analysis due to the unavailability of suitable biopsy and plasma samples that had been collected prior to the initiation of treatment. As shown in Table S1, 10 patients provided informed consent; hence were included in this study. Our inclusion criteria necessitated that the participants be pathologically confirmed to have recurrent/persistent OSCC after either surgery, concurrent chemoradiotherapy, or systemic chemotherapy with/without radiotherapy. For the whole‐exome sequencing (WES) analysis, we selected five of the 10 patients with OSCC who have three positive OSCC (positive for the expression of PD‐L1 and CD8^+^ T cells), one weak positive OSCC (weak positive for the expression of PD‐L1 and CD8^+^ T cells), and one negative OSCC (negative for the expression of PD‐L1 and CD8^+^ T cells).

### 2.2 Nivolumab or pembrolizumab therapy

Patients with recurrent/persistent OSCC received 240 mg nivolumab intravenously every 2 weeks or 480 mg every 4 weeks. Or they received 200 mg pembrolizumab intravenously every 3 weeks until disease progression or unacceptable toxic effects occur.

### 2.3 Immunohistochemistry (IHC)

Ten samples were suitable for the assessment of PD‐L1 expression. PD‐L1 expression was assessed using the PD‐L1 IHC (CST13684; dilution; 1:200). CPS was calculated by summing the number of PD‐L1‐stained cells; the number of positive cells in each sample was calculated after the analysis of five high‐power fields (400×).

For the CD8 IHC test, cells were incubated with anti‐CD8 (DAKO 144B; dilution; 1:100). CD8^+^ T‐cell density was quantitatively assessed. After the intratumoral (IT) CD8^+^ T cells were identified in the five locations at low magnification, they were counted manually in the areas of highest CD8^+^ intensity (400×), and cell counts were averaged.

Assessment of both PD‐L1 and CD8 staining was accurately performed by three pathologists at our institution.

### 2.4 WES and variant interpretation

To screen for genetic variants associated with OSCC, we collected a pair of blood serum samples before treatment and matched formalin‐fixed paraffin‐embedded tumor samples from five of the 10 patients with OSCC for WES. The genomic regions of whole exons were captured by microarray using synthesized oligonucleotide probes hybridized to a fragmented genomic DNA sample. The libraries were sequenced with 2 × 150 bp paired‐end reads using an Illumina NovaSeq 6000 instrument. Exome enrichment was performed using a SureSelect Human All Exon V6 kit (Agilent, USA), according to the manufacturer's instructions, and the sequences were compared with the human reference genome b37. Briefly, raw sequencing data in the FASTQ format were aligned against the reference human genome (b37) using Samtools. The Genome Analysis Toolkit and Sentieon tools were used for germline single‐nucleotide variant and indel calling, whereas the Sentieon tool was used for somatic single‐nucleotide variant and small indel calling. ANNOVAR was used to functionally annotate genetic variants.

## 3 RESULTS

### 3.1 Analysis of PD‐L1 and CD8 expression

**FIGURE 1:**
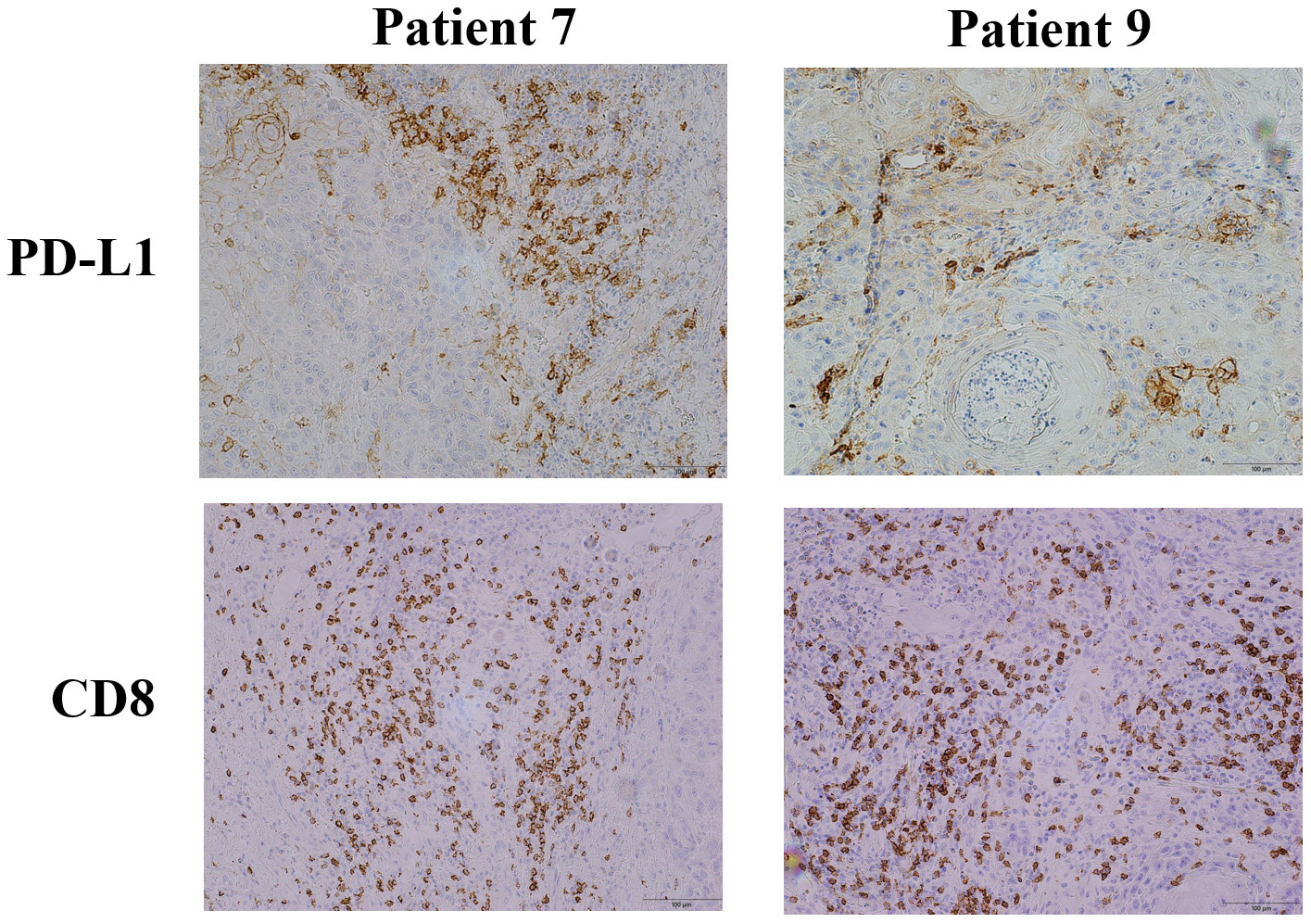
Histopathological features of biopsy tissue obtained from patients with OSCC.

As previously described, the IHC findings were integrated for each patient, and a total of 10 samples were eligible for the assessment of PD‐L1 expression and intratumoral microscopic fields, as illustrated in Figure 1. Based on the CPS, seven (70%) patients were described to be positive (CPS >1%), including five (50%) and two (20%) patients with a CPS >20% and 1% ≤ CPS ≤20%, respectively.

Furthermore, IT CD8^+^ T‐cell infiltration was strong positive (>50%) in four (40%) and weak positive (<50%) in four (40%) samples and negative (<1%) in two (20%) samples. As shown in Figure 1, Patients 7 and 9 were both positive for PD‐L1 and CD8^+^ T.

### 3.2 Analysis of somatic mutation by WES

**FIGURE 2:**
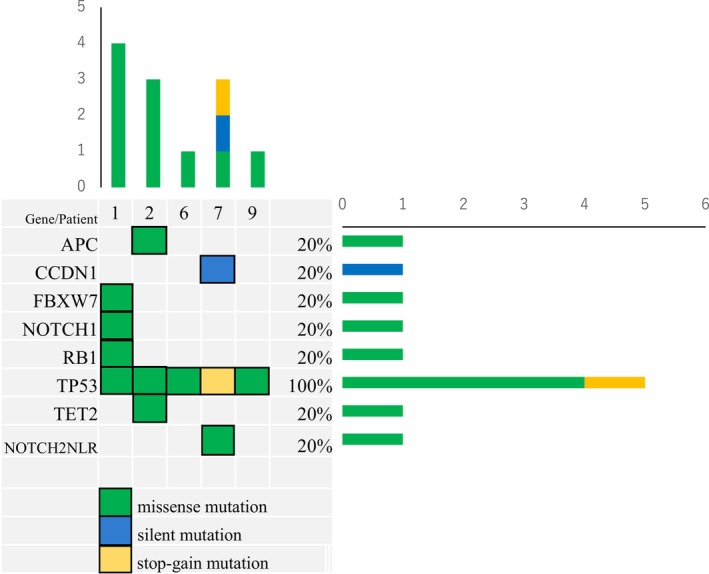
Oncoplot depicts gene mutations identified from biopsy tissue by whole‐exome sequencing (WES). Heatmap summarizing mutational signature, somatic variants derived from WES of patient tumors.

To investigate the impact of somatic mutations on ICI response, we analyzed gene expression and WES data. The different somatic mutations observed in five patients with OSCC are illustrated in Figure 2.

Specifically, Patient 1 exhibited missense mutations in the NOTCH1 and FBXW7 genes, whereas Patient 7 exhibited a missense mutation in the NOTCH2NLR gene. Notably, we could not detect the mutation of NOTCH2NLR gene in posttreatment sample of Patient 7, as indicated in Table S2.

### 3.3 Analysis of the treatment duration

Ten patients received various therapies prior to initiating ICI treatment. The median duration of treatment for our study population was 43.7 months, with a range of 18–140 months.

**FIGURE 3:**
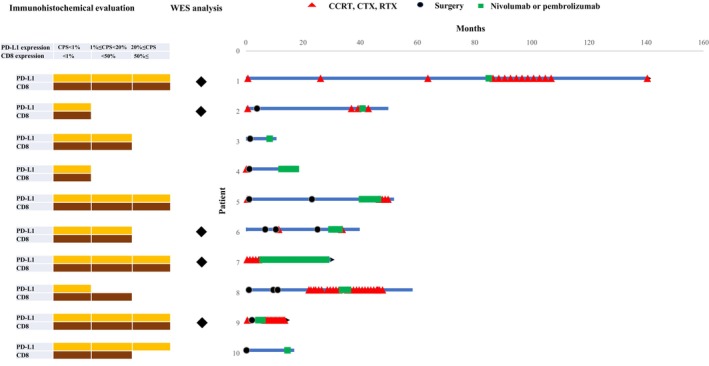
Swimmer plot of treatment duration of all study participants. Swimmer plot indicating response to and duration of therapy for individual patients. The timing response and the period of surgery are also indicated. The red triangle indicates concurrent chemoradiotherapy (CCRT), chemotherapy (CTX) or radiotherapy (RTX). The black dot indicates surgery. The green square indicates nivolumab or pembrolizumab administration.

For one patient who achieved a PR, the time to for clinical response after the administration of ICI was 26 months. Among the six patients who demonstrated SD, the median time from treatment initiation to the initial response was 23.6 months, with a range of 12–58 months. Additionally, for the two patients with PD, the median duration of clinical response was 128 months, ranging from 58 to 140 months. The swimmer plots in Figure 3 (*n* = 10) provide a detailed visualization of our patients' overall treatment durations.

### 3.4 irAE

There were two treatment‐related AEs in 10 patients: One case of both myocarditis and myositis after two cycles of pembrolizumab, whereas the other case of complicated chronic heart failure after one cycle of nivolumab. Due to this, treatment discontinuation was required for these two patients (Patient 3 and Patient 10 in Table S1).

## 4 DISCUSSION

As per reports, there is recent evidence that Notch signaling pathways are often likely to cause tumorigenesis and promotion of OSCC.^11^ Mutations of genes in the Notch signaling pathway have been reported in solid tumors.^12^ In our retrospective study, we analyzed somatic mutations by WES. Notably, Patient 1, harboring missense mutations in both *Notch1* and *FBXW7*, Patient 7, with a missense mutation in*NOTCH2NLR* gene, and Patient 9, lacking missense mutations in Notch signaling genes, all exhibited an increased CD8^+^ T‐cell expression. However, Patient 9 appeared to have no effect on antitumor immunogenic responses, indicating that mutations in certain genes, aside from TP53, may contribute to antitumor immunogenic response. Notch signaling genes, including *Notch1, FBXW7*, and *Notch 2* may be important factors in Notch signaling pathway. *Notch2* is regulated mutually with *Notch1*. *NOTCH2NLR* (NOTCH2NL‐related) mutations were found to be present in the primary tumor.^13^ This remarkable gene is located near *NOTCH2* on the p‐arm of chromosome 1. The >100‐kb genomic regions spanning each *NOTCH2NL* gene show >99.1% sequence identification for *Notch2*.

While at the same time, we confirmed the expression of PD‐L1, which was very high in both Patient 1 and 7 (with Notch pathway mutations) as well as Patient 9 (without Notch pathway mutations), along with the infiltration of CD8^+^ T cells. It has been reported that tumor‐specific CD8^+^ T cells infiltrate around PD‐1^+^ cells, which also exhibit increased expression of mucin‐domain‐containing‐3 (TIM‐3) and lymphocyte activation gene‐3 (LAG3).^14^ Additionally, Wang et al.'s original articles have demonstrated that gene mutations within the Notch signaling pathway can exhibit the antitumor immunogenic roles of CD8^+^ T cells and impair the inhibitory effects of regulatory T cells.^15^ These findings suggest that Patients 1 and 7 may exhibit signs of exhaustion in the tumor‐specific CD8^+^ T‐cell subset, indicating a potential target for cancer cells. Moreover, a high level of Tumor‐associated neutrophils (TANs) is also associated with a worse long term survival and immunological tolerance in epithelial ovarian cancer. TANs partially impair the cytotoxic roles of CD8^+^ T cells through Jagged2 (JAG2). When the Notch pathway is suppressed by an inhibitor, such as a γ‐secretase inhibitor, treatment with an anti‐JAG2 antibody results in the attenuation of tumor growth and augmentation of cytotoxic roles of CD8^+^ T cells.^16^

In the present study, we aimed to elucidate whether PD‐L1 upregulation caused CD8^+^ T‐cell infiltration in aberrant Notch signaling pathway. The present study has several limitations. First, a small number of patients with OSCC exhibited aberrant Notch signaling and the underlying mechanism of PD‐L1 upregulation is not fully understood. Second, specific domains essential for the functional loss of NICD is unclear. In currently ongoing in vitro and clinical studies, we are conduction comprehensive analyses to delineate the mechanism of CD8+ T cells infiltration mediated by PD‐L1 upregulation.

Interestingly, several OSCC cell lines exhibit aberrant Notch1 expression. Some of these cell lines harbor Notch1 mutations despite the increased expression of nuclear PD‐L1. On the other hand, nuclear PD‐L1 was not expressed in a cell line which did not harbor aberrant Notch1 expression. It is possible that tumor cells with aberrant Notch1 signaling acquire unidentified oncogenic functions, which lead to increased PD‐L1 expression in the nucleus. Therefore, to confirm the induction in PD‐L1 expression and CD8^+^ T‐cell infiltration at the protein level, we plan to obtain consent and perform immunohistochemical analysis to evaluate PD‐L1 expression and CD8^+^ T‐cell infiltration in patients with OSCC harboring *Notch1* mutations, who were reported in a previous study.^17^

In summary, in the present study we found a potential relationship between mutations in the Notch signaling pathway and CD8 T^+^ cell infiltration via PD‐L1 expression, leading to enhanced antitumor immunity, which might confer clinical benefit to patients with recurrent or metastatic OSCC on ICI therapy.

## AUTHOR CONTRIBUTIONS

**Kazuhiro Ogi:** Conceptualization (lead); data curation (equal); formal analysis (equal); writing – original draft (equal); writing – review and editing (equal). **Takahiro Iwamoto:** Data curation (equal); formal analysis (equal); methodology (equal). **Takashi Sasaya:** Data curation (equal); formal analysis (equal); methodology (equal). **Koyo Nishiyama:** Data curation (equal); methodology (equal). **Takaaki Tokura:** Data curation (equal). **Takanori Sasaki:** Data curation (equal). **Hironari Dehari:** Data curation (equal). **Yohei Arihara:** Data curation (equal). **Kazuyuki Murase:** Data curation (equal). **Masato Saito:** Data curation (equal). **Masanori Someya:** Supervision (equal); writing – review and editing (equal). **Kohichi Takada:** Supervision (equal); writing – review and editing (equal). **Akihiro Miyazaki:** Supervision (equal); writing – review and editing (equal).

## FUNDING INFORMATION

Japan Society for the Promotion of Science, Grant‐Number: JSPS KAKENHI 22K10197.

## CONFLICT OF INTEREST STATEMENT

There is no conflict of interest regarding this study.

## ETHICS STATEMENT

This study was authorized by the Institutional Review Board of Sapporo Medical University (reference No. 332‐3410 from the Sapporo Medical University Committee).

## PATIENT CONSENT STATEMENT

All patients gave written informed consent.

## Supporting information


Table S1.

Table S2.

## Data Availability

The data that support the findings of this study are available on request from the corresponding author.
